# A Three-Step Synthesis of Squalene from *trans, trans*-Farnesol

**DOI:** 10.1021/acsomega.6c06150

**Published:** 2026-08-14

**Authors:** Marcel Achtenhagen, Thomas Silldorff, Kit H. B. Foster, Tayler Barone, Rudrajit Mal, Evon Bolessa, Alexis Ellis, Raman Bahulekar, Nandkumar Deorkar, Erik J. Sorensen

**Affiliations:** † Avantor, Inc., 77 Corporate Drive, Bridgewater, New Jersey 08807, United States; ‡ Department of Chemistry, 6740 Princeton University, Princeton, New Jersey 08544, United States

## Abstract

A three-step synthesis of squalene (**1**), the universal precursor of sterols, an adjuvant for vaccines, and a component of cosmetics, has been achieved from plant-derived *trans, trans*-farnesol (**2**) and 2-nitrobenzenesulfonamide (**3**). The successful strategy described herein features an end-to-end pairing of farnesyl radicals formed in one laboratory operation from bis-farnesylamine (**6**) and Levin’s anomeric amide (**7**). This concise synthesis provides squalene in 99.8% purity after purification of the derived thiourea clathrate, following the procedure of Nicolaides and Laves.

## Graphical abstract

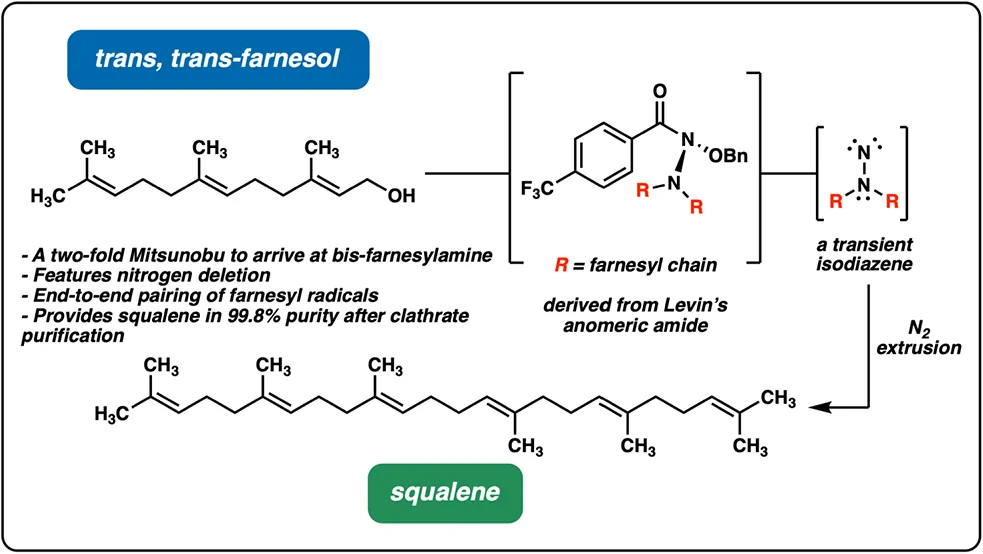

## Introduction

In 1916, Tsujimoto discovered that a hydrocarbon oil with a molecular formula of C_30_H_50_ is the major constituent of the liver oils of squaloid sharks and proposed the name *squalene* for the highly unsaturated hydrocarbon.^4^ During the period of 1926–1929, Heilbron and coworkers reported the outcomes of their systematic study of the chemical reactions of squalene, which allowed them to deduce its structure, shown as compound (**1**), Figure 1.^5–7^ Squalene is the universal precursor of the polycyclic triterpenes, including cholesterol, cholesterol-derived steroid hormones, and the large class of plant- and bacterial-derived hopanoids.^8–11^ It is also an important component of adjuvant formulations for vaccines, health supplements, nutraceuticals, and cosmetics.^12–17^ The demand for squalene as an adjuvant in vaccines is especially high and anticipated to grow as the cell-gene therapy sector continues to evolve.^18–21^

**Figure 1:**
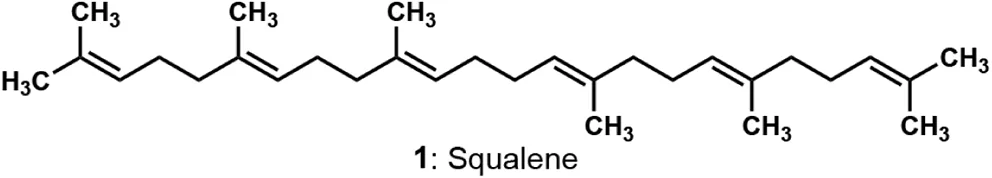
Molecular structure of squalene (**1**).

There are serious concerns over the sustainability of obtaining vaccine-grade squalene from shark liver oil, which has exposed an urgent need for alternative, shark-free sources of squalene. One promising avenue for producing high-purity squalene on a large scale is organic synthesis. However, the synthetic process must meet quality, scalability, and cost-effectiveness attributes to be a viable alternative. To date, more than 30 laboratory syntheses of squalene have been described,^3,22–47^ including the recent 5-step semisynthesis of squalene from farnesene by Fox, Paddon, and McPhee.^48^ The latter achievement is particularly significant because it is a GMP synthesis capable of furnishing vaccine-grade squalene on kilogram scales. Herein, we describe our recent 3-step synthesis of squalene from *trans,trans*-farnesol (**2**). This synthesis features the generation and end-to-end pairing of transient farnesyl carbon radicals and is capable of yielding squalene in a high state of purity.^49^

## Results and Discussion

A 2-fold Mitsunobu substitution of plant-derived *trans, trans*-farnesol (**2**) with 2-nitrobenzenesulfonamide (**3**), mediated by tetramethyl-azo-dicarboxamide (**4**) and tri-*n*-butylphosphine, afforded *bis*-farnesylsulfonamide (**5**) in 79% yield after workup (Scheme 1). The subsequent cleavage of the *o*-nitrobenzenesulfonamide in (**5**) by reaction with the thiolate ion derived from thioglycolic acid according to the method of Fukuyama and co-workers^50,51^ was also clean and furnished *bis*-farnesylamine (**6**) in 74% yield and in a sufficiently high state of purity after workup, without the need for a chromatographic purification step. Our strategy required a method that would be capable of excising the nitrogen atom in (**6**) with a concomitant coupling of the farnesyl chains. Of the potential methods for achieving this objective, the method of Levin and co-workers^1^ was especially attractive, and we were delighted to observe that the reaction of *bis*-farnesylamine (**6**) with Levin’s anomeric amide (**7**) in THF at 45 °C is capable of producing squalene (**1**) via hydrazine derivative (**8**) and the putative isodiazene (**9**). The irreversible loss of nitrogen gas from the transient isodiazene (**9**) leaves in its wake two farnesyl radicals that preferentially dimerize in an end-to-end fashion to produce crude squalene (**1**) in 36% yield. To the best of our knowledge, we believe that the low yield of desired squalene is due to the competitive formation of a 1,2-diazene rather than a 1,1-diazene. This arises from a competitive [2,3]-sigmatropic rearrangement of the 1,1-diazene, resulting in the formation of isomers of squalene after extrusion of nitrogen. For best results, it is essential that the pivalic acid used in the preparation of Levin’s anomeric amide (**7**) be free of water.

**Scheme 1:**
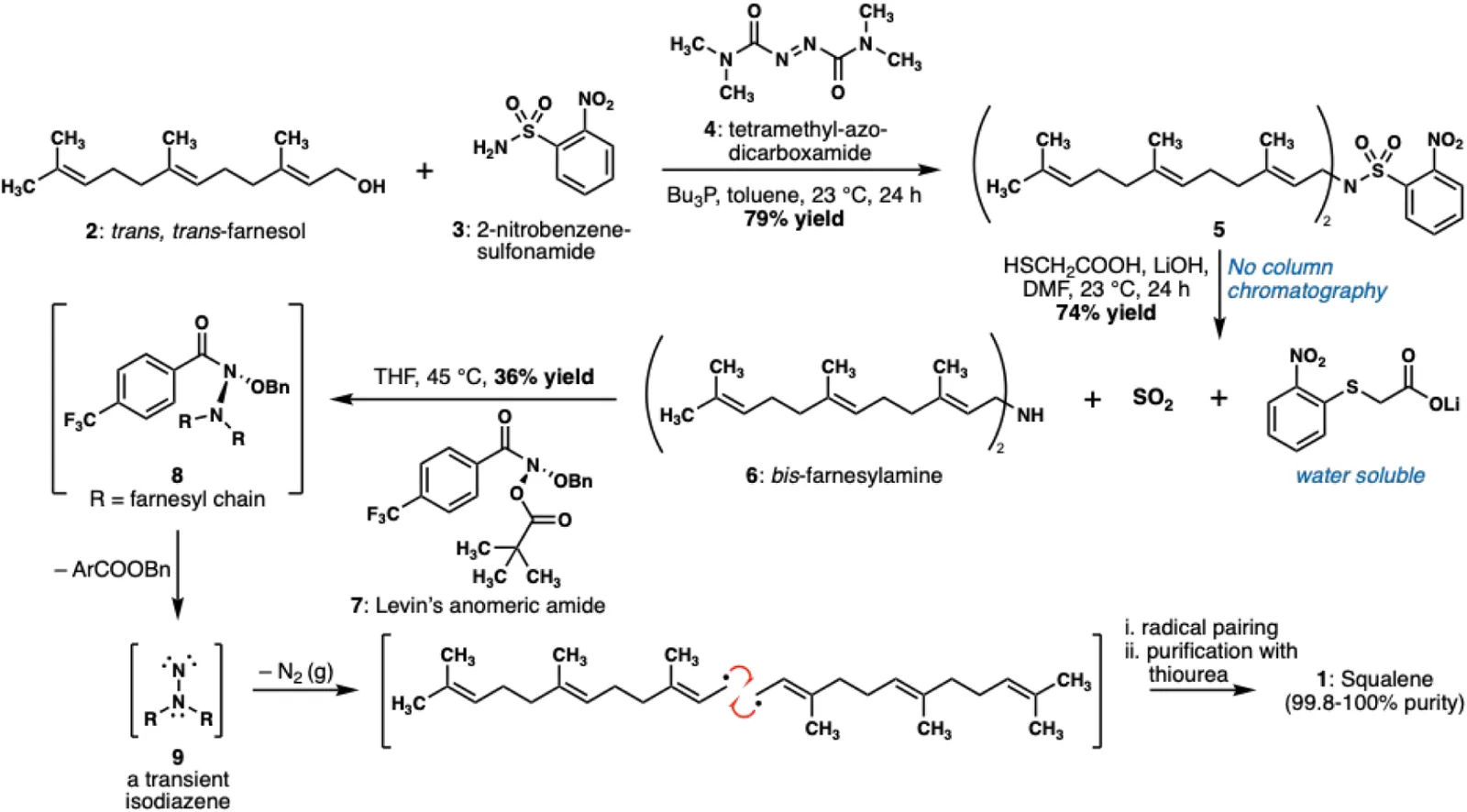
A Three-Step Synthesis of Squalene (**1**) from *trans, trans*-Farnesol (**2**) Featuring a Pairing of Farnesyl Radicals

At the outset, we were worried that the allylic farnesyl radicals generated in this reaction might combine haphazardly and yield squalene as a component of complex mixtures. It was thus gratifying to discover that the pairing of solvent-caged farnesyl radicals to produce squalene could be a relatively clean process. Following the procedures of Nicolaides and Laves^2^ and the Cornforth laboratory,^3^ crude squalene (36%) produced in this fashion was treated with thiourea. It was straightforward to free the resulting clathrates from impurities. A subsequent water workup and extraction of the hydrocarbon with petroleum ether afforded synthetic squalene (**1**) with a purity of 99.8–100% and in 29% yield from bis-farnesylamine (**6**).

## Conclusions

From inexpensive, plant-derived *trans,trans*-farnesol (**1**), a concise three-step synthesis of the versatile and important polyunsaturated hydrocarbon squalene (**1**) was achieved in 29% overall yield by an approach featuring a dimerization of farnesyl radicals. A pleasing aspect of the approach described herein is that one of the three steps of the synthesis furnishes an intermediate that does not require purification. Efforts are underway to improve the yields of each of the three steps, adapt this synthesis to the production of squalene on large scales, and reduce the overall cost of the process.

## Experimental Section

### General Protocols

All reactions were performed in flame-dried glassware with magnetic stirring and under an atmosphere of dry argon or nitrogen. ACS reagent-grade chloroform (CHCl_3_), dichloromethane (CH_2_Cl_2_), *N,N*-dimethylformamide (DMF), ethyl acetate (EtOAc), hexanes, methanol (CH_3_OH), petroleum ether, and tetrahydrofuran (THF) were purchased and used without additional purification. Anhydrous toluene (PhCH_3_) and THF were purchased in septum-sealed bottles and dried further over 4Å molecular sieves. All reactions were monitored by thin layer chromatography (TLC) using glass plates with a 0.25 mm layer of silica gel and a fluorescent indicator. TLC plates were visualized by exposure to UV light and stained with either acidic ethanolic para-anisaldehyde solution or basic potassium permanganate solution. Silica gel for flash column chromatography was 230–400 mesh with a 40–63 μm particle size and a 60 Å pore size. Proton nuclear magnetic resonance (^1^H NMR) spectra were collected at 400 MHz and calibrated to the residual monoprotio solvent peak (CHCl_3_: 7.26 ppm). Coupling constants were extracted assuming first-order coupling, and peak multiplicities are abbreviated as follows: s = singlet, d = doublet, t = triplet, q = quartet, quint = quintet, m = multiplet, br = broad signal, and app = apparent signal. Proton-decoupled carbon nuclear magnetic resonance (13C­{1H} NMR) spectra were measured at 100 MHz and calibrated to the deuterated solvent peak (CDCl_3_: 77.16 ppm). Infrared (IR) spectra for reaction products were recorded using an ATR accessory. High-resolution mass spectral (HRMS) data were obtained using either an electrospray ionization (ESI+) LC/MS or an electron impact (EI) GC/MS equipped with a time-of-flight mass analyzer. Elemental analysis data were obtained using a Velp model EMA 502 elemental microanalyzer with a combustion oven temperature of 1030.0 °C and a GC oven temperature of 55.0 °C.

*2-Nitro-N-((2E,6E)-3,7,11-trimethyldodeca-2,6,10-trien-1-yl)-N-((2Z,6Z)-3,7,11-trimethyldodeca-2,6,10-trien-1-yl)­benzenesulfonamide* (**5**)

Commercial *trans, trans*-farnesol (**2**) (4.39 g, 19.8 mmol, 2.00 eq., 96% purity, Sigma-Aldrich) was added to a flame-dried 100 mL round-bottom flask containing 2-nitrobenzenesulfonamide (**3**) (2.00 g, 9.89 mmol, 1.00 equiv). The atmosphere in the flask was exchanged three times with argon, and then anhydrous toluene (29.96 mL) was added. The flask was cooled to 0 °C using an ice bath. Then, tri-*N*-butylphosphine (7.41 mL, 29.67 mmol, 2.20 equiv) was added, followed by the addition of 1,1’-azobis­(*N,N*-dimethylformamide) (**4**) (5.11 g, 29.67 mmol, 3.00 equiv). The reaction was allowed to stir at 0 °C for 10 min, after which it was allowed to warm to 23 °C and was stirred at this temperature for 24 h. After 24 h, toluene was removed under reduced pressure on a rotary evaporator at 30 °C to yield a brown crude oil, which was diluted with H_2_O (100 mL) to yield an aqueous mixture that was extracted with hexanes (3 × 50 mL). The organic layer was washed with H_2_O (2 × 100 mL) and saturated aqueous sodium chloride (100 mL) before drying over anhydrous sodium sulfate. The solvent was removed under reduced pressure on a rotary evaporator at 30 °C to yield a brown oil. The crude oil was filtered through a pad of silica gel using hexanes as the eluent (250 mL) and then switching to 20% EtOAc:hexanes (500 mL) and collecting the EtOAc:hexanes fraction. The solvent was removed under reduced pressure on a rotary evaporator at 30 °C to afford sulfonamide (**5**) as a brown oil (4.76 g, 7.79 mmol, 79%).

^1^H NMR (500 MHz, CDCl_3_): δ 8.03 (dd, *J* = 7.2, 2.0 Hz, 1H), 7.69–7.60 (m, 3H), 5.07 (m, 6H), 3.90 (d, *J* = 6.9 Hz, 4H), 2.08–1.92 (m, 16H), 1.68 (s, 6H), 1.61–1.56 (m, 18H); ^13^C NMR (126 MHz, CDCl_3_): δ 148.2, 141.0, 140.0, 135.6, 134.6, 133.2, 131.6, 131.5, 131.1, 124.5, 124.4, 124.2, 123.9, 123.7, 123.5, 119.0, 59.6, 44.5, 39.8, 39.7, 39.7, 26.9, 26.4, 26.4, 25.8, 17.8, 16.4, 16.3, 16.2; IR (ATR) v 2915, 1544, 1439, 1371, 1349, 1160, 1125, 913, 851, 759, 736, 583 cm^–1^ ; HRMS (ESI+) calcd for C_36_H_54_N_2_O_4_S ([M + H]^+^): 611.3877, found 611.3886, calcd for C_36_H_54_N_2_O_4_SNa ([M + Na]^+^): 633.3696, found 633.3718. Elemental analysis: calcd for C_36_H_54_N_2_O_4_S: %C = 70.78, %H = 8.91, %N = 4.59, %O = 10.48, %S = 5.25, found: %C = 72.4804, %H = 9.1405, %N = 4.0468, %O = 10.8478, %S = 5.1259.

*(2Z,6Z)-3,7,11-Trimethyl-N-((2E,6E)-3,7,11-trimethyldodeca-2,6,10-trien-1-yl)­dodeca-2,6,10-trien-1-amine* (**6**)

Sulfonamide (**5**) (0.50 g, 0.82 mmol, 1.00 equiv) was added to a 25 mL round-bottom flask, and then solid lithium hydroxide (0.078 g, 3.28 mmol, 4.00 equiv) was added. To this, DMF (2.00 mL) was added, followed by thioglycolic acid (0.15 g, 1.64 mmol, 2.00 equiv), and the reaction was allowed to stir under a nitrogen atmosphere (nitrogen balloon) at 23 °C for 24 h. After 24 h, the reaction was diluted with saturated aqueous sodium bicarbonate (10 mL) and H_2_O (10 mL), followed by extraction with hexanes (3 × 20 mL). The organic layer was washed with H_2_O (3 × 100 mL) and saturated aqueous sodium chloride (100 mL) before drying over anhydrous sodium sulfate. The solvent was removed under reduced pressure on a rotary evaporator at 40 °C to yield a brown oil. The crude oil was dissolved in hexanes (10 mL) and partitioned with acetonitrile (10 mL) and swirled for 30 s. The bottom acetonitrile layer was removed, and fresh acetonitrile (10 mL) was added and swirled again for 30 s. This process was repeated one more time, after which the top hexane layer was removed under reduced pressure on a rotary evaporator at 40 °C to afford (**6**) as a brown oil (0.259 g, 0.61 mmol, 74%).

^1^H NMR (500 MHz, CDCl_3_): δ 5.27 (t, 2H), 5.15–5.06 (m, 4H), 3.22 (d, *J* = 6.8 Hz, 4H), 2.13–1.94 (m, 16H), 1.68 (s, 6H), 1.64 (s, 6H), 1.60 (s, 12H); ^13^C NMR (126 MHz, CDCl_3_): δ 137.8, 135.3, 131.4, 124.5, 124.2, 124.2, 123.2, 46.9, 39.9, 39.9, 39.8, 26.9, 26.9, 26.6, 25.8, 17.8, 16.5, 16.1; IR (ATR) 2966, 2915, 2853, 1668, 1442, 1378, 1151, 1108, 984, 833, 741, 451 cm^–1^; HRMS (ESI+) calcd for C_30_H_51_N ([M + H]^+^): 426.4094, found 426.4216. Elemental analysis: calcd for C_30_H_51_N: %C = 84.64%, %H = 12.07, %N = 3.29, found: %C = 84.6502%, %H = 12.0091%, %N = 2.9541.

*(6E,10E,14E,18E)-2,6,10,15,19,23-Hexamethyltetracosa-2,6,10,14,18,22-hexaene* (**1**)

Amine (**6**) (5.00 g, 11.74 mmol, 1.00 equiv) was charged into a flame-dried 100 mL amber round-bottom flask, and the atmosphere was exchanged with argon three times. To this was added anhydrous and degassed THF (45 mL), and the solution was heated to 45 °C. In a separate flame-dried vial, Levin’s anomeric amide (**7**) (6.96 g, 17.61 mmol, 1.50 equiv) was added to anhydrous and degassed THF (15 mL). This solution was added quickly in one portion to the 100 mL amber round-bottom flask at 45 °C and stirred at this temperature for 16 h. After 16 h, the reaction was allowed to cool to 23 °C and was then quenched with saturated aqueous sodium bicarbonate (10 mL) and extracted with hexanes (3 × 50 mL). The organic layer was dried over anhydrous sodium sulfate. The solvent was removed under reduced pressure on a rotary evaporator at 25 °C to yield a pale-yellow oil. The crude oil was dissolved in hexanes (100 mL) and washed with saturated aqueous sodium bicarbonate (3 × 100 mL) and H_2_O (3 × 100 mL) before drying over anhydrous sodium sulfate. The solvent was removed under reduced pressure on a rotary evaporator at 25 °C to yield a pale-yellow oil. The crude oil was dissolved in hexanes (100 mL) and filtered through a pad of silica gel using hexanes (500 mL) as the eluent. The solvent was removed under reduced pressure on a rotary evaporator at 28 °C to afford crude squalene (**1**) as a clear colorless oil (1.73 g, ∼36%). NMR analysis revealed crude squalene (**1**) to be the major compound, with a purity in the range of 85–90%.

### Clathrate Purification to Yield 99.9% Pure Squalene

Crude squalene (**1**) (1.73 g, 4.21 mmol, 1.00 equiv) was dissolved in a solution of thiourea (39.8 g, 522.86 mmol, 124.19 equiv) in methanol (310 mL), and this solution was cooled to 0 °C for 1 h. After 1 h, the crystals that formed were collected and washed with petroleum ether (3 × 20 mL) and then air-dried under a stream of nitrogen gas. The dried crystals were added to H_2_O (30 mL) to hydrolyze the clathrate. The aqueous layer was extracted with petroleum ether (3 × 30 mL) before drying the organic layer over anhydrous sodium sulfate. The solvent was removed under reduced pressure on a rotary evaporator at 25 °C to afford squalene (**1**) as a clear colorless oil (1.4 g, 3.41 mmol, 29%, purity of 99.8–100% by GC).

^1^H NMR (500 MHz, CDCl_3_): δ 5.19–5.07 (m, 6H), 2.16–1.95 (m, 20H), 1.69 (s, 6H), 1.61 (s, 18H); ^13^C NMR (126 MHz, CDCl_3_): δ 135.2, 135.0, 131.4, 124.6, 124.5, 124.4, 39.9, 39.9, 28.4, 26.9, 26.8, 25.8, 17.8, 16.2, 16.1; IR (ATR) 2966, 2914, 2853, 1442, 1381, 1376, 1329, 1150, 1107, 984, 834, 451 cm^–1^. Elemental analysis: calcd for C_30_H_50_: %C = 87.73, %H = 12.27, found: %C = 87.6706, %H = 12.3149.

## Supplementary Material



## Notes

General protocols and analytical methods, as well as high-resolution NMR spectra (PDF)
