# Supplementary material for: A Three-Step Synthesis of Squalene from trans, trans-Farnesol
Source: ACS Omega. 2026 Aug 14;11(33):50303–7. doi: 10.1021/acsomega.6c06150 (PMC13520434; doi:10.1021/acsomega.6c06150)
Supplement: Supplementary file 1 [file ao6c06150_si_001.pdf]

A Three-step Synthesis of Squalene from *trans*, *trans*-Farnesol

Marcel Achtenhagen[1], Thomas Silldorff[2], Kit H. B. Foster[2], Tayler Barone[1], Rudrajit Mal[1], Evon Bolessa[1], Alexis Ellis[1], Raman Bahulekar[1*], Nandkumar Deorkar[1*] and Erik J. Sorensen[2*]

[1]Avantor, Inc., 77 Corporate Drive, Bridgewater, New Jersey 08807 and [2]Department of Chemistry, Princeton University, Princeton, New Jersey 08544, United States

[*]ejs@princeton.edu

Table of Contents

General Procedures and Analytical Methods S2

NMR Spectra S3

General Protocols and Analytical Methods

All reactions were performed in flame-dried glassware with magnetic stirring and under an atmosphere of dry argon or nitrogen. ACS reagent grade chloroform ($CHCl_3$), dichloromethane (CH_2Cl_2), *N,N*-dimethylformamide (DMF), ethyl acetate (EtOAc), hexanes, methanol (CH_3OH), petroleum ether, and tetrahydrofuran (THF), were purchased and used without additional purification. Anhydrous toluene ($PhCH_3$) and THF, were purchased in septum-sealed bottles and dried over 4Å molecular sieves. All reactions were monitored by thin layer chromatography (TLC) using glass plates with a 0.25 mm layer of silica gel and a fluorescent indicator. TLC plates were visualized by exposure to UV light and stained with either acidic ethanolic para-anisaldehyde solution or basic potassium permanganate solution. Silica gel for flash column chromatography was 230-400 mesh with a 40–63 μm particle size and a 60 Å pore size. Proton nuclear magnetic resonance (^{1}H NMR) spectra were collected at 500 MHz and are calibrated to the residual monoprotio solvent peak ($CHCl_3$: 7.26 ppm). Coupling constants were extracted assuming first-order coupling, and peak multiplicities are abbreviated as follows: s = singlet, d = doublet, t = triplet, q = quartet, quint = quintet, m = multiplet, br = broad signal, and app = apparent signal. Proton-decoupled carbon nuclear magnetic resonance (^{13}C{^{1}H} NMR) spectra were measured at 126 MHz and calibrated to the deuterated solvent peak ($CDCl_3$: 77.16 ppm). Infrared (IR) spectra for reaction products were recorded using an ATR accessory. High resolution mass spectral (HRMS) data were obtained using either an electrospray ionization (ESI+) LC/MS or an electron impact (EI) GC/MS equipped with a time-of-flight mass analyzer. Elemental analysis data were obtained using Velp model EMA 502 elemental microanalyzer with a combustion oven temperature of 1030.0 °C and a GC oven temperature of 55.0 °C.

NMR Spectra

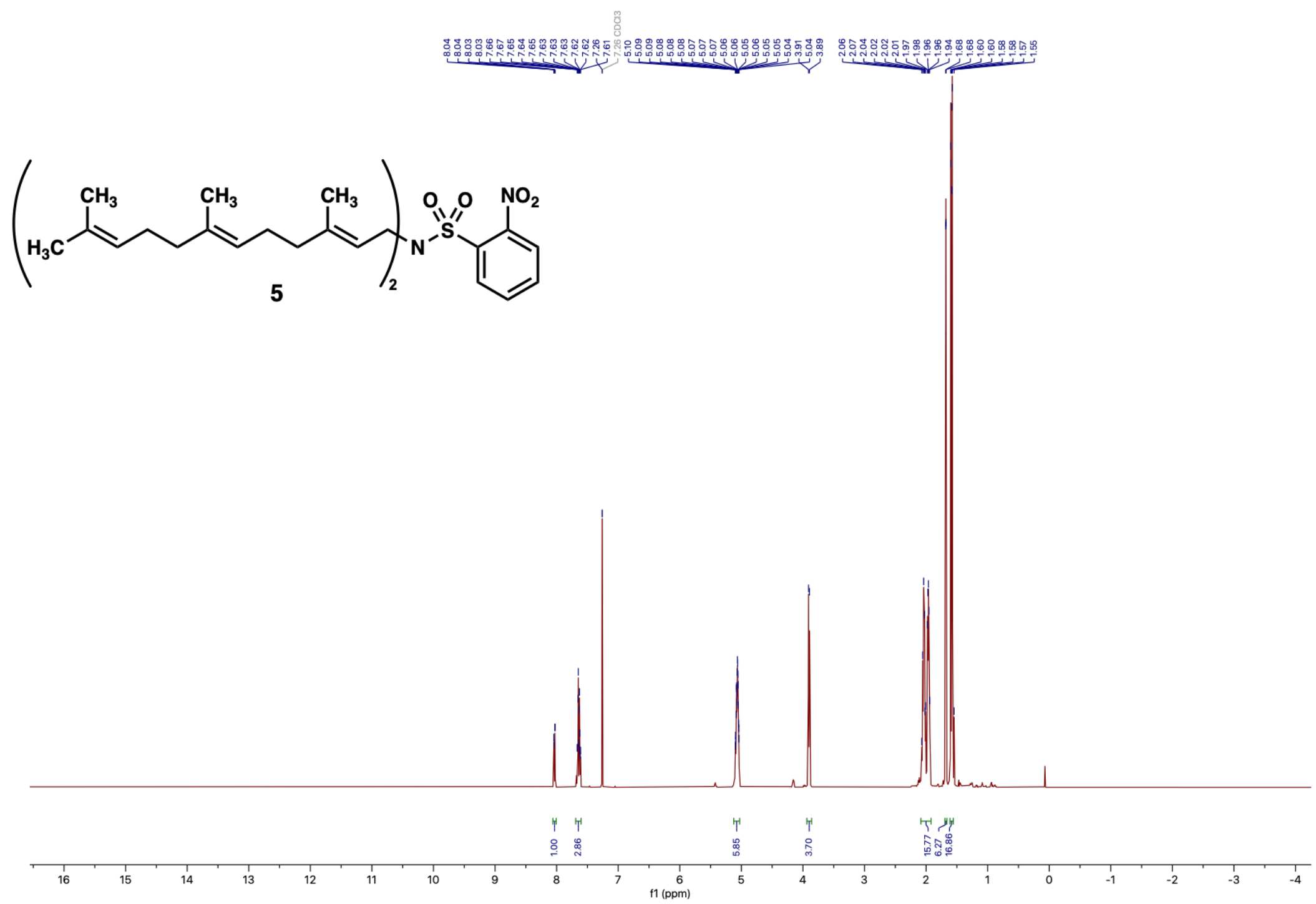

Figure S1: ^{1}H-NMR (500 MHz, CDCl$_3$) of compound **5**

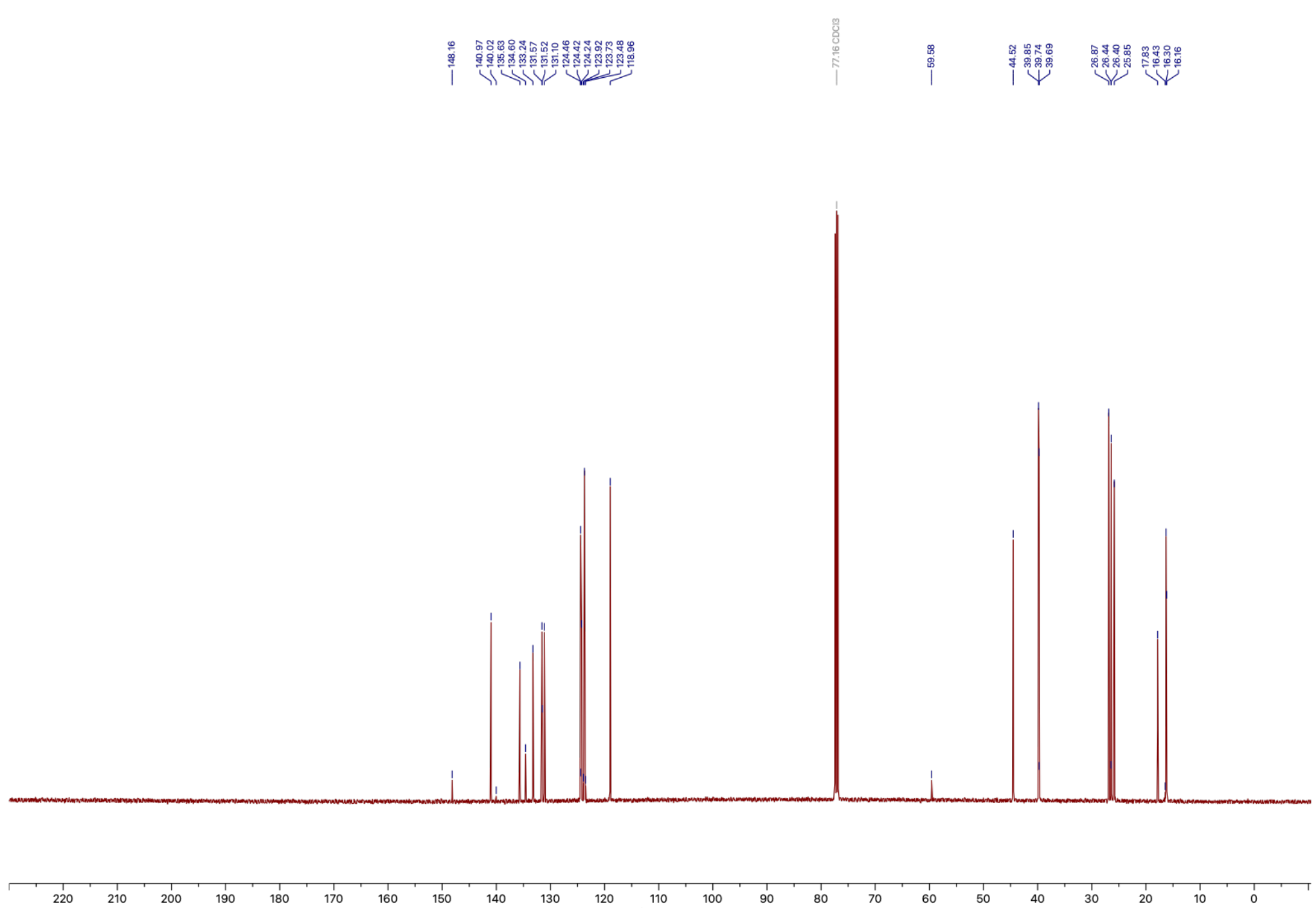

Figure S2: ^{13}C{^{1}H} NMR (126 MHz, CDCl$_3$) of compound **5**

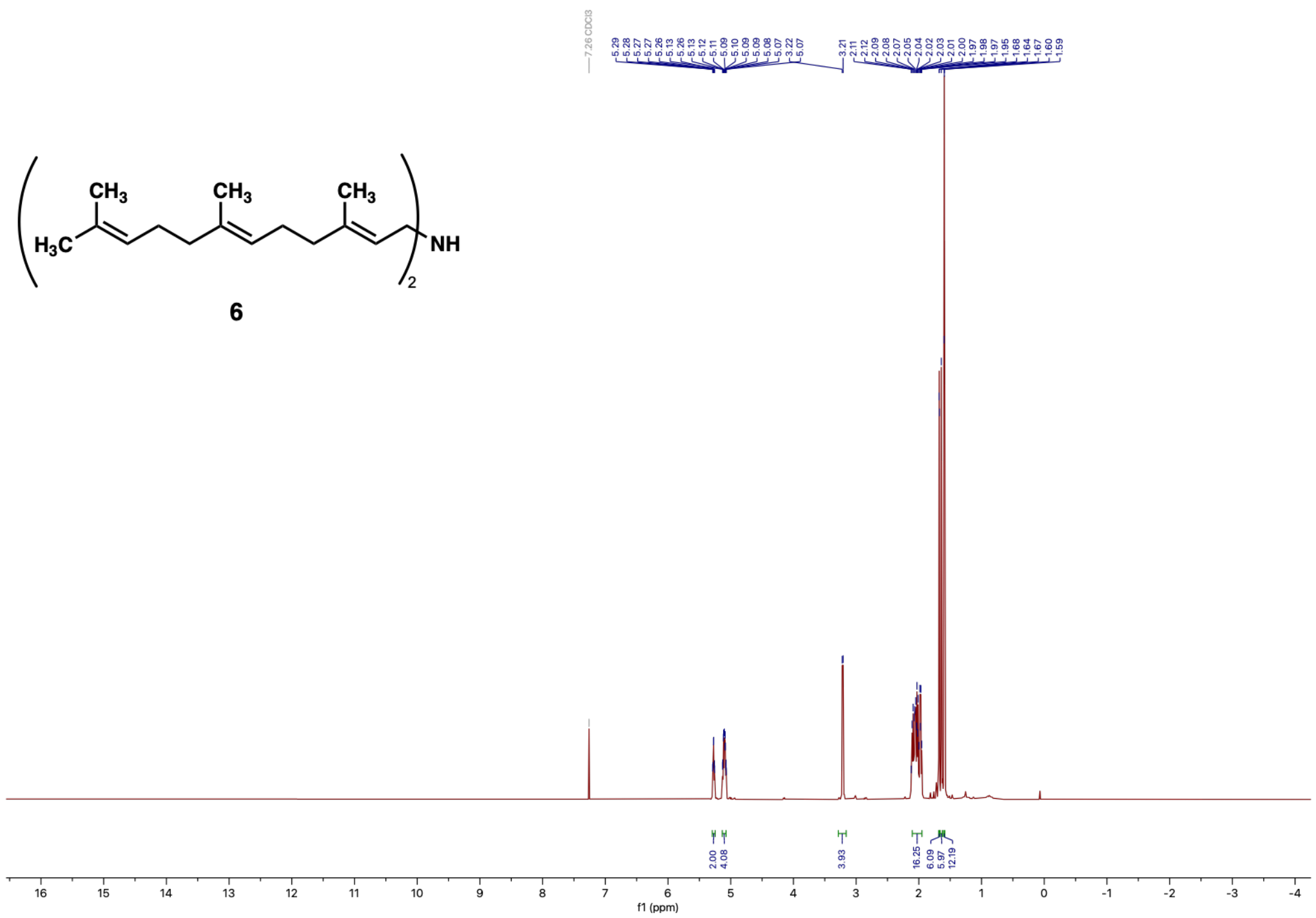

Figure S3: ^{1}H-NMR (500 MHz, $CDCl_3$) of compound **6**

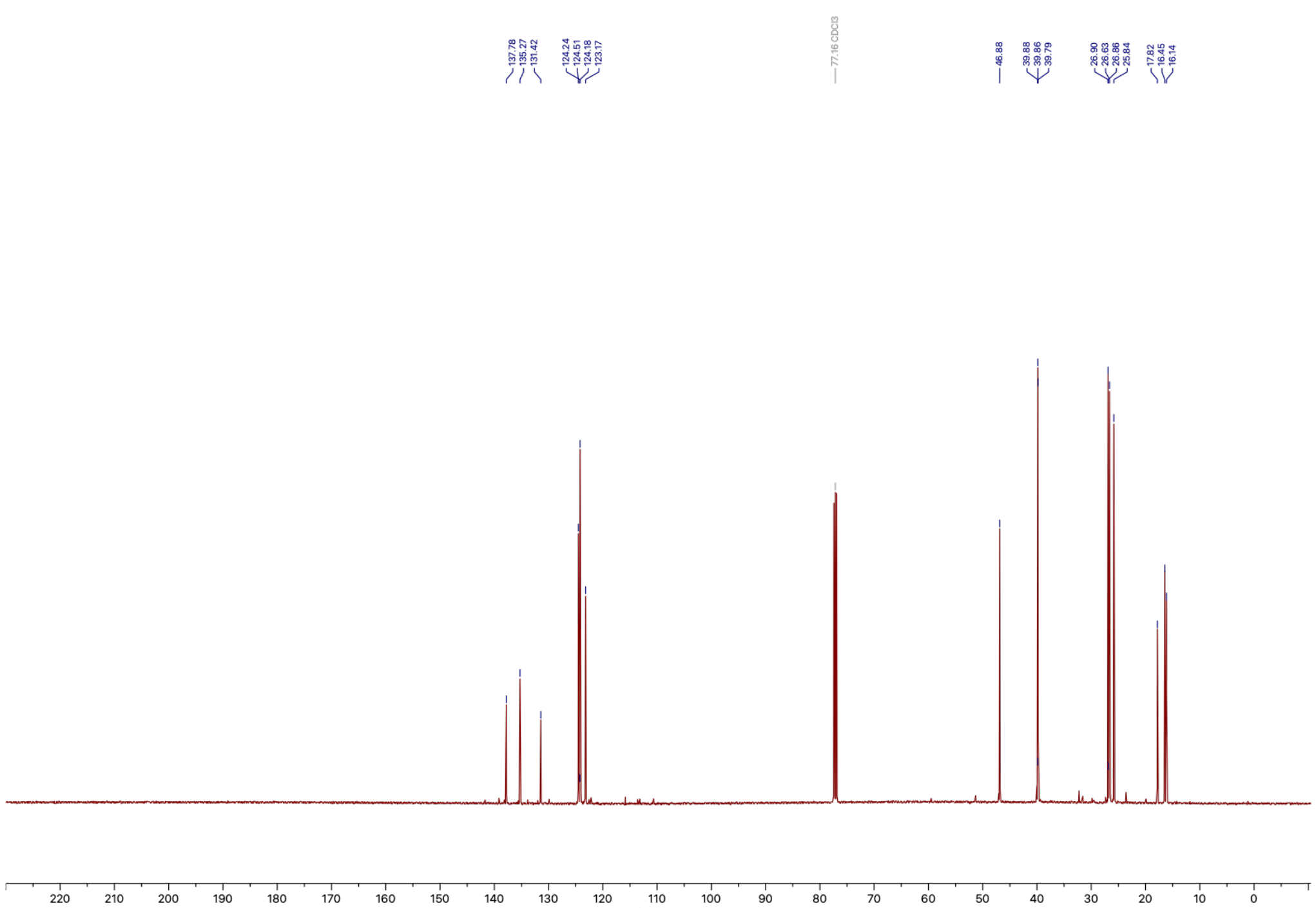

Figure S4: $^{13}C\{^{1}H\}$ NMR (126 MHz, $CDCl_3$) of compound **6**

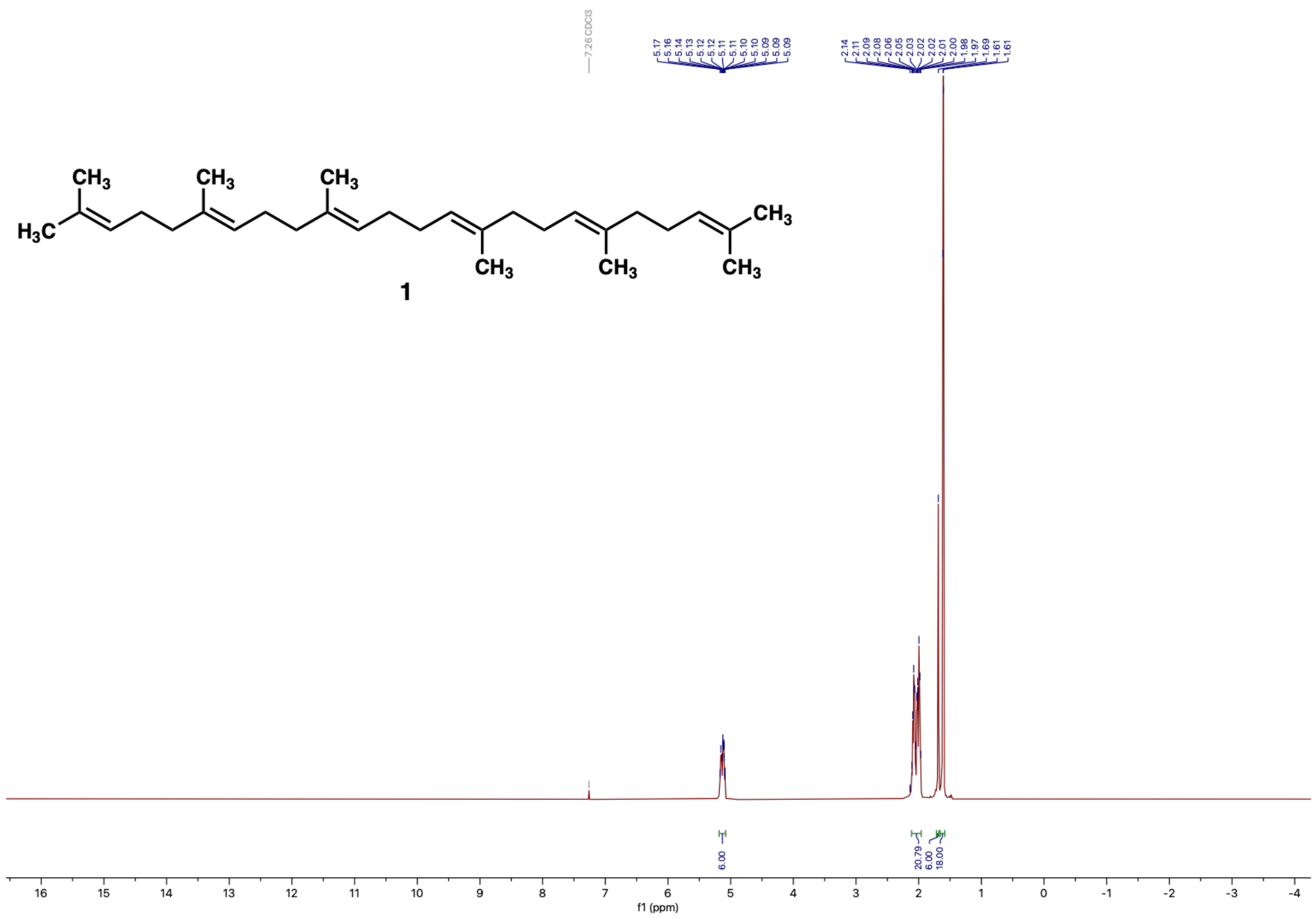

Figure S5: ^{1}H-NMR (500 MHz, $CDCl_3$) of compound **1**

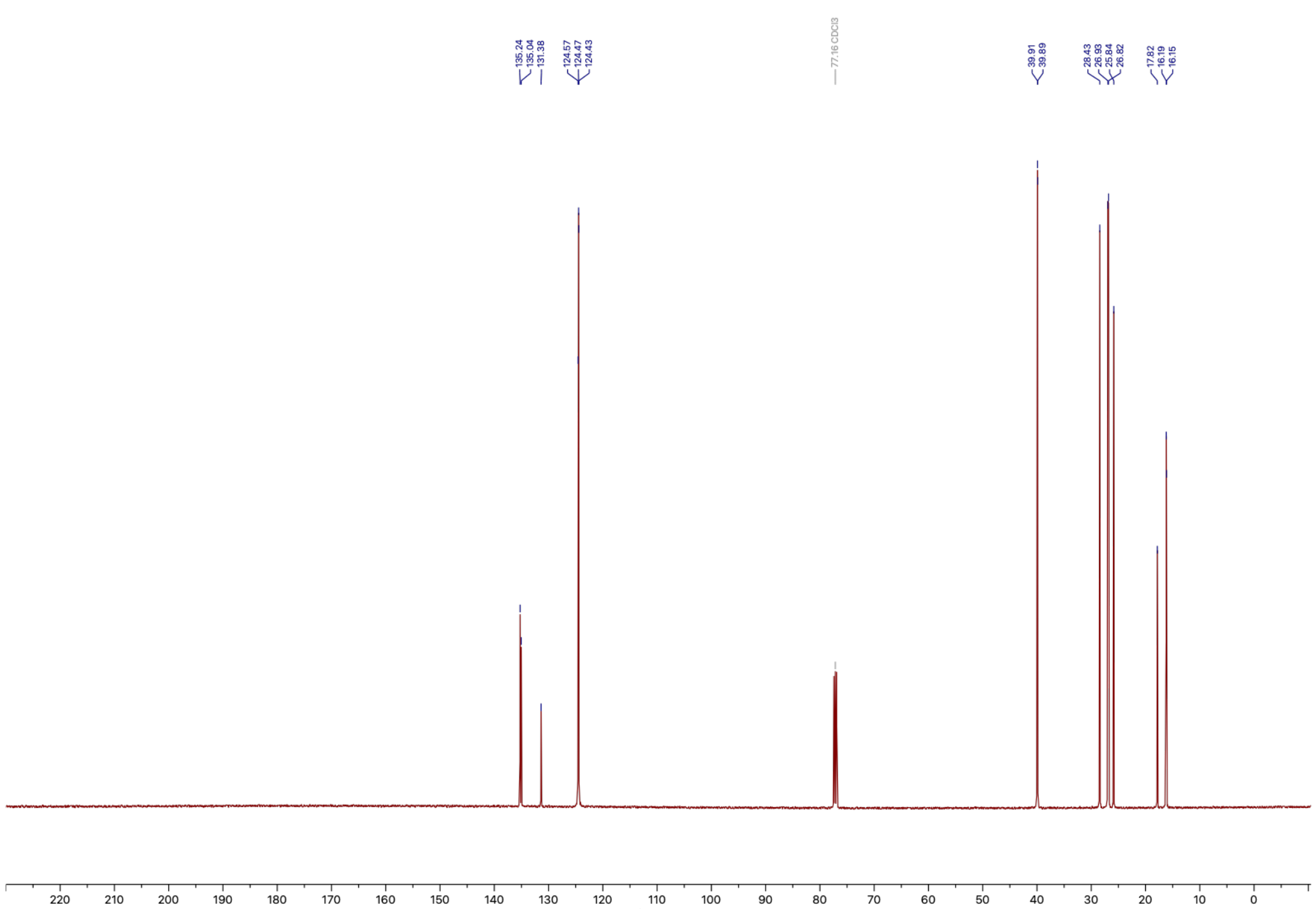

Figure S6: $^{13}C\{^{1}H\}$ NMR (126 MHz, $CDCl_3$) of compound **1**
